# Data preparation techniques for a perinatal psychiatric study based on linked data

**DOI:** 10.1186/1471-2288-12-71

**Published:** 2012-06-08

**Authors:** Fenglian Xu, Lisa Hilder, Marie-Paule Austin, Elizabeth A Sullivan

**Affiliations:** 1 Perinatal and Reproductive Epidemiology Research Unit, School of Women and Children’s Health, University of New South Wales, Randwick, NSW 2031, Australia; 2 Perinatal & Women's Mental Health Unit, St John of God Health Care & School of Psychiatry, University of New South Wales, Burwood, NSW 2134, Australia

**Keywords:** Data preparation, Method, Psychiatric study, Australia

## Abstract

**Background:**

In recent years there has been an increase in the use of population-based linked data. However, there is little literature that describes the method of linked data preparation. This paper describes the method for merging data, calculating the statistical variable (SV), recoding psychiatric diagnoses and summarizing hospital admissions for a perinatal psychiatric study.

**Methods:**

The data preparation techniques described in this paper are based on linked birth data from the New South Wales (NSW) Midwives Data Collection (MDC), the Register of Congenital Conditions (RCC), the Admitted Patient Data Collection (APDC) and the Pharmaceutical Drugs of Addiction System (PHDAS).

**Results:**

The master dataset is the meaningfully linked data which include all or major study data collections. The master dataset can be used to improve the data quality, calculate the SV and can be tailored for different analyses. To identify hospital admissions in the periods before pregnancy, during pregnancy and after birth, a statistical variable of time interval (SVTI) needs to be calculated. The methods and SPSS syntax for building a master dataset, calculating the SVTI, recoding the principal diagnoses of mental illness and summarizing hospital admissions are described.

**Conclusion:**

Linked data preparation, including building the master dataset and calculating the SV, can improve data quality and enhance data function.

## Background

Existing population and hospital data are valuable sources of surveillance and research. Data linkage provides a tangible route to make the data sources more powerful for research [1,2]. In recent years, there has been a marked increase in the use of registry data for births, deaths and diseases such as psychiatric disorders [3,4]. Hospital admission data has become one of the key data collections for medical research especially for population-based cohort studies[5]. In New South Wales (NSW), the Midwives Data Collection (MDC), the Registry of Births, Deaths and Marriages (RBDM) registration data, the Admitted Patient Data Collection (APDC) and the Cancer Registry (CR) are commonly used by medical researchers for their studies [6-9]. However, there is little literature that describes the method of linked data preparation. Research articles using linked data did not describe data preparation details in method section because of the word limit. Method papers about linked data mainly focused on strategies rather than on techniques [2,5,10]. However, the data preparation techniques including syntax are often inquired by researchers.

This paper aims to answer four frequently asked questions in data preparation using linked birth and hospital data. The first question is how are data collections merged? The second is how are hospital admissions distributed into the periods before pregnancy, during pregnancy and after birth? The third is how are psychiatric diagnoses recoded and grouped? The fourth is how are hospital admissions summarised and ordered in sequence?

### Data sources

Data sources used in this paper as examples are: The NSW Midwives Data Collection (MDC) which includes mothers’ and babies’ records, the NSW Register of Congenital Conditions (RCC), the NSW Admitted Patient Data Collection (APDC) and the NSW Pharmaceutical Drugs of Addiction System (PHDAS). The MDC is a population-based data collection of all births in NSW. It covers all births of at least 20 weeks gestation or at least 400 grams birthweight in public and private hospitals and homebirths. It includes information on maternal characteristics, pregnancy, labour, delivery and neonatal outcomes. The NSW Department of Health has managed the MDC since 1987. Personal identifiers were included in the data collection after 1992 and there was a revision to the MDC form in 1993. It is recommended that record linkage studies be carried out on data collected since 1994. The RCC is a population-based surveillance system and monitors birth defects detected during pregnancy, at birth or up to one year after birth. The data is available for a rolling five-year period. It covers structural birth defects but not functional problems. The RCC was established in 1990. As the RCC was voluntary up to 1997, the birth defects records were incomplete especially for terminations of pregnancy. Since 1998, the reporting of terminations of pregnancy has improved and doctors, hospitals and laboratories have been required to notify the register of all birth defects. The APDC is a routinely collected census of all hospital separations. It includes all patient hospitalisations from NSW public, private and repatriation hospitals, private day procedures centres and public nursing homes. The data include patient demographics, diagnoses and clinical procedures. The APDC is collected on a financial year basis. A limitation of APDC data is that there were no names in the APDC prior to 1 July 2000. Since 1 July 2000, the APDC has included names for patients admitted to public hospitals but does not have names for patients admitted to private hospitals. Other information such as sex, date of birth, medical record number, hospital code and address can be used to assist in linkage. The PHDAS is an administrative database used by the Pharmaceutical Services Unit of the NSW Ministry of Health to facilitate the authorisation of medical practitioners to prescribe drugs of addiction. It consists of the Methadone Subsystem implemented in 1985, the Non-Methadone Subsystem implemented in 1985 and the Stimulant Notification Subsystem implemented in 1999. This study used the NSW Opioid Treatment Program (OTP) in the Methadone Subsystem and included treatment information such as program type, start and end date, reason for ending program and other drugs of concern.

Data linkage was performed by The Centre for Health Record Linkage (CHeReL) of the NSW Department of Health using probabilistic record linkage methods and choicemaker software (http://www.cherel.org.au). Each record was assigned a record identification number, the Project Person Number, which allows the records for the same individual to be identified, extracted and linked. The CHeReL Checked a random sample of 1,000 persons, the false positive rate of the linkage was 0.3% and false negative <0.5%.

The study was approved by the NSW Population & Health Services Research Ethics Committee and the Human Research Ethics Committee of the University of New South Wales.

### Study design

The aims of this study are: to investigate the pattern and rate of hospital admission for maternal psychiatric disorders and substance use before and after birth; explore the factors associated with these problems; and compare the perinatal outcomes of babies whose mothers were admitted or not admitted to hospital for mental illness before birth.

This study was based on linked data which include the MDC, RCC, APDC and PHDAS. The study population included all mothers aged from 18 to 44 years who gave birth between 1 January 2003 and 31 December 2004 in NSW, and their babies. Birth records from 2003 to 2004 in the MDC were linked with the RCC in the same period and with APDC and PHDAS records between 1 January 2001 and 31 December 2006. This enabled hospital admissions for the mothers to be traced back to at least two years before birth and followed up for at least two years after birth. Each mother was followed up from the year before pregnancy to the end of the 24th month after birth.

The key variables included in the study included Project Person Number for mother and baby; mother’s age in days at birth; mother’s age in days at hospital admission and discharge; diagnoses for psychiatric disorders, substance use and birth defects; maternal age; mother’s country of birth; maternal diabetes mellitus and hypertension; pregnancy complications (pre-eclampsia, gestational diabetes); smoking status during pregnancy; remoteness of living area and a socioeconomic indicator (i.e. the Index of Relative Socio-economic Disadvantage Quintile); delivery method; infant gender; birthweight; gestational age; admission to a neonatal intensive care unit (NICU) or special care nursery (SCN); and fetal/neonatal death type (stillbirth, neonatal death, post-neonatal death). The reason for using mother’s age in days at birth, at hospital admission and discharge rather than date of birth, hospital admission and discharge is to maintain privacy and decrease the risk of inadvertent identification of individuals from the data.

The main measurements for mothers were hospital admission rate of maternal psychiatric disorders and substance use before pregnancy, during pregnancy and after birth; and factors associated with maternal psychiatric disorders and substance use. The main pregnancy outcomes included birthweight, preterm birth, birth defects and admission to an NICU or SCN. The outcomes were compared between babies whose mothers were admitted and not admitted to hospital with the diagnoses of mental illness.

The diagnoses in hospital data were classified using ICD-10-AM (International Statistical Classification of Diseases and Related Health Problems Tenth Revision, Australian Modification). The diagnoses included principal, stay and all diagnosis. The principal diagnosis, coded ‘icd10d1’, referred to a medical condition that was chiefly responsible for the hospital admission [11]. An additional diagnosis, coded from ‘icd10d2’ to ‘icd10d55’, was a condition or a complaint either coexisting with the principal diagnosis or arising during the hospitalization. Stay diagnosis was an additional diagnosis coded ‘icd10d2’ and referred to the diagnosis that most influenced the length of stay in hospital. The stay diagnosis was frequently the same as the principal diagnosis; however, it may be different [12].

### Merge data

**Figure 1:**
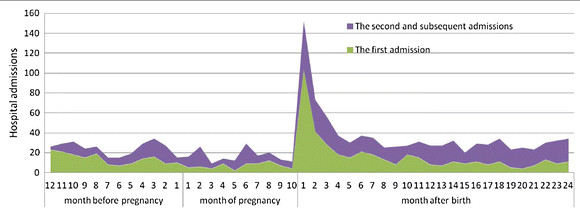
The hospital admissions of low prevalent psychiatric disorders before and after birth.

For studies based on linked data collections, there are generally two ways to merge the data. One is to link all or the majority of study datasets to build a master dataset. Then the master dataset can be used for variable preparation and tailored into sub data for different study purposes. There are two advantages of building a master dataset. One is that it provides a platform to improve the data quality. The consistency of values for variables that repeatedly appear in the master dataset can be checked and completeness improved [13]. For example, the variable mother’s country of birth (Cob) appeared in each birth record of the MDC; if the mother had two births, her Cob could be checked for consistency and the missing value could be minimised. Maternal Indigenous status was under-reported in the NSW births[14,15]. Maternal Aboriginal status was under-ascertained in both the MDC and RBDM. By linking the MDC and RBDM and constructing an SV of Aboriginality, the number of missing values of Aboriginality was significantly decreased and the under-estimation was significantly improved [13]. Another advantage is that the master dataset provides an opportunity to calculate an SV which identifies hospital admissions in the periods before pregnancy, during pregnancy and after birth (such as Admmonth in Table1). The SV of time interval (SVTI) can also be used to select study populations. For example, the mothers who admitted to hospital with a diagnosis of mental illness in the period from 12 months before pregnancy to 24 months after birth could be selected using the SVTI for the analysis of rates before and after birth (see Figure1). Another way to merge data is to select study records from different data collections according to study topic and merge these to build the study data for the specific analysis. The advantage of this method is that the merged data size is relatively small which makes the merge easier.

**Table 1:** The steps and SPSS syntax to calculate and recode the statistical variable of Admmonth

Step	Variable and label	SPSS syntax	Explanation
1	Admdays: number of days between hospital admission and birth.	COMPUTE Admdays = AGEAdmMum – AgeBirthMum.VARIABLE LABELS Admdays 'Hospital admission days before and after birth'.EXECUTE.	If the value of Admdays is less than 0, it means the hospital admission is before birth.A positive value means the admission is after birth.
2	Admmonth: admission month before pregnancyGage : gestational age in days	IF (Admdays < 0) Admmonth = Admdays + Gage.EXECUTE.RECODE Admmonth (lowest thru −361 = 0)(−360 thru −331 = 1)(−330 thru −301 = 2)……(−60 thru −31 = 11) (−30 thru −1 = 12).EXECUTE.	Value 0 refers to the admission before the 12^th^ month before pregnancy.1: the admission in the 12^th^ month before pregnancy……11: the admission in the second month before pregnancy.12: the admission in the first month before pregnancy.
3	Admmonth: admission month during pregnancy	RECODE Admmonth (0 thru 29 = 13) (30 thru 59 = 14)…… (300 thru 329 = 23).EXECUTE.	13: the admission in the first month of pregnancy.14: the admission in the second month of pregnancy……23: the admission in the 11^th^ month of pregnancy.
4	Admmonth: admission month after birth	DO IF (Admdays > = 0).RECODE Admdays (0 thru 29 = 24**)** (30 thru 59 = 25**)……** (330 thru 359 = 35**)** (360 thru Highest = 36) INTO Admmonth.END IF.EXECUTE.VARIABLE LABELS Admmonth 'Hospital admission month before and after birth'.VALUE LABELS Admmonth 0 'before' 1 ' the 12th month before pregnancy' ……11 ' the 2nd month before pregnancy' 12 ' the 1st month before pregnancy’ 13 ' the first month of pregnancy ' 14' the second month of pregnancy '……23' the 11^th^ month of pregnancy' 24 ' the first month after birth ' 25' the second month after birth '……35' the 12^th^ month after birth '36' after'.	24: refers to the admission in the first month after birth.25: refers to the admission in the second month after birth……35: refers to the admission in the 12^th^ month after birth.36: refers to the admission after the 12^th^ month after birth.

**Figure 2:**
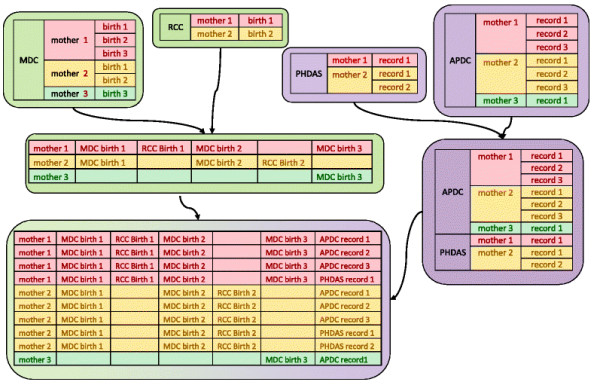
The data merged on MDC,RCC, mothers’ APDC and PHDAS.

An example of merging a master dataset for a maternal mental illness study is shown in Figure2. Firstly, the birth records in the MDC were merged with the records in the RCC for the same birth. Secondly, the birth records of one mother were merged by birth order of the mother into a single record. Finally, the mother’s linked birth data were merged with the APDC and PHDAS (see Figure2) by mother’s Project Person Number. The master dataset included all available information for the study and could be tailored for different analyses.

### Prepare the variable which distributes hospital admissions over the periods before and after birth

In order to describe the trend of hospitalisations before and after birth (see Figure1), an SVTI needs to be created. The SVTI can identify hospital admissions over different time intervals such as a week, month or year, before and after birth. It also shows the time of admission before and during pregnancy. The steps and SPSS syntax for calculating and recoding an SVTI variable, Admmonth, which identifies hospital admissions during the months before and after birth, are shown in Table1. The Admmonth is calculated by three variables: maternal age in days at birth in the MDC (AgeBirthMum); mother’s age in days at admission in the APDC (AGEAdmMum); and gestational age in days (Gage). Gestational age was provided from the MDC as the number of completed weeks since the start of the last menstrual period. As a result, it needs to be converted into days to keep the unit consistent with the previous two variables. Because the Gage is reported in completed weeks, the accuracy of hospital admission time before birth is also in weeks rather than in days. For interval recoding, each month is defined as 30 days and each year as 360 days.

The hospital admissions in other time intervals such as year (Admyear) can be calculated the same way. The only difference is the length of the interval recoded. For example, each value of Admyear covers 360 days while Admmonth covers 30 days.

### Classify diagnoses of mental illness

Mental illnesses are generally classified into diagnoses groups according to study purpose, psychiatric definition and sample size. As a result, the International Classification of Diseases (ICD) code in each category may vary across research papers [16-19]. Table2 shows the diagnostic categories or groupings used for our study and SPSS syntax which allows recoding of the principal diagnoses of mental illness into these groups. The most prevalent diagnoses found in this study are those represented in the table: unipolar depression (including major depression F32 codes and those depressions identified as arising in the first six weeks after birth F53.0 code); bipolar disorder; acute or brief psychotic episodes; adjustment and anxiety disorders; and schizophrenia and schizophrenia-like disorder. In addition we are interested in substance use disorders — there is a category for less common remaining disorders (e.g. somatoform disorders) as well as an overall mental illness category where all diagnoses are grouped together.

**Table 2:** Classification of principal diagnoses for mental illness

Category	ICD-10-AM	Diagnosis	SPSS syntax
Prin1: Schizophrenia and schizophrenia-like disorder	F20	Schizophrenia	COMPUTE Prin1 = 0.if ( icd10d1 GE 'F20' AND icd10d1 LE 'F22.9' ) Prin1 = 1.if ( icd10d1 GE 'F24' AND icd10d1 LE 'F29' ) Prin1 = 1.EXECUTE.VARIABLE LABELS Prin1' principal diagnoses of Schizophrenia and schizophrenia-like disorder F20-22, F24-29'.
	F21	Schizotypal disorder	
	F22	Persistent delusional disorders	
	F24	Induced delusional disorder	
	F25	Schizoaffective disorders	
	F28	Other nonorganic psychotic disorders	
	F29	Unspecified nonorganic psychosis	
Prin2: Unipolar depressions	F32	Depressive episode except F32.3 see below (psychotic).	COMPUTE Prin2 = 0.if ( icd10d1 GE 'F32' AND icd10d1 LE 'F32.21' ) Prin2 = 1.if ( icd10d1 GE 'F32.8' AND icd10d1 LE 'F32.91' ) Prin2 = 1.if ( icd10d1 EQ 'F53.0' ) Prin2 = 1.if ( icd10d1 EQ 'F34.1' ) Prin2 = 1.if ( icd10d1 EQ 'F38.1' ) Prin2 = 1.if ( icd10d1 EQ 'F38.8' ) Prin2 = 1.EXECUTE.VARIABLE LABELS Prin2 ' principal diagnoses of unipolar depressions F32 (exclude 32.3), F53.0, F34.1, F38 (exclude 38.0)'.
	F53.0	Mild disorders associated with the puerperium, not elsewhere classified	
	F34.1	Persistent mood [affective] disorders: Dysthymia	
	F38	Other mood [affective] disorders except F38.0 see below (bipolar affective episode)	
Prin3: Acute Psychotic episodes : reactive, brief, affective	F23	Acute and transient psychotic disorders	COMPUTE Prin3 = 0.if ( icd10d1 GE 'F23' AND icd10d1 LE 'F23.91' ) Prin3 = 1.if ( icd10d1 GE 'F32.3' AND icd10d1 LE 'F32.31' ) Prin3 = 1.if ( icd10d1 EQ 'F30.2' ) Prin3 = 1.if ( icd10d1 EQ 'F33.3' ) Prin3 = 1.if ( icd10d1 EQ 'F39' ) Prin3 = 1.if ( icd10d1 EQ 'F53.1' ) Prin3 = 1.EXECUTE.VARIABLE LABELS Prin3' principal diagnoses of acute psychotic episodes: reactive, brief, affective F23, 30.2,32.3, 33.3, 39, 53.1'.
	F30.2	Mania with psychotic features	
	F32.3	Severe depressive episode with psychotic symptoms	
	F33.3	Recurrent depressive disorder, current severe with psychotic symptoms	
	F39	Unspecified mood [affective] disorder	
	F53.1	Severe MH disorders associated with the puerperium, not elsewhere classified	
Prin4: Bipolar affective disorders	F30.0	Hypomania	COMPUTE Prin4 = 0.if ( icd10d1 GE 'F30.8' AND icd10d1 LE 'F30.9' )Prin4 = 1.if ( icd10d1 GE 'F31' AND icd10d1 LE 'F31.6' ) Prin4 = 1.if ( icd10d1 GE 'F31.8' AND icd10d1 LE 'F31.9' ) Prin4 = 1.if ( icd10d1 EQ 'F30.0' ) Prin4 = 1.if ( icd10d1 EQ 'F34.0' ) Prin4 = 1.if ( icd10d1 EQ 'F38.0' ) Prin4 = 1.EXECUTE.VARIABLE LABELS Prin4' principal diagnoses of bipolar affective disorders F30 (exclude 30.2), 31(exclude 31.7),34.0,38.0 '.
	F30.8	Other manic episodes	
	F30.9	Manic episode, unspecified	
	F31	Bipolar affective disorder F31.0-31.9 excluding F31.7 (remission).	
	F34.0	Persistent mood [affective] disorders: Cyclothymia	
	F38.0	Other single mood [affective] disorders	
Prin5: Adjustment disorders	F43	Reaction to severe stress and adjustment disorders	COMPUTE Prin5 = 0.if ( icd10d1 GE 'F43' AND icd10d1 LE 'F43.9' ) Prin5 = 1.EXECUTE.VARIABLE LABELS Prin5' principal diagnoses of adjustment disorders F43 '.
Prin6: Anxiety disorders	F40	Phobic anxiety disorders	COMPUTE Prin6 = 0.if ( icd10d1 GE 'F40' AND icd10d1 LE 'F42.9' ) Prin6 = 1.EXECUTE.VARIABLE LABELS Prin6' principal diagnosis of anxiety disorders F40-42'.
	F41	Other anxiety disorders	
	F42	Obsessive-compulsive disorder	
Prin7: Personality disorders	F60-69	Personality disorders	COMPUTE Prin7 = 0.if ( icd10d1 GE 'F60' AND icd10d1 LE 'F69' ) Prin7 = 1.EXECUTE.VARIABLE LABELS Prin7' principal diagnoses of personality disorders F60-69'.
Prin8: Mental illness due to substance use	F10-F19	Mental illness due to substance use	COMPUTE Prin8 = 0.if ( icd10d1 GE 'F10' AND icd10d1 LE 'F19.8' ) Prin8 = 1.EXECUTE.VARIABLE LABELS Prin8 ' principal diagnoses of mental illness due to substance use F10-19. '.
Prin9: Remaining diagnoses	F00-99 Exclude the diagnoses above	Remaining diagnoses	COMPUTE Prin9 = 0.if ( icd10d1 GE 'F00' AND icd10d1 LE 'F09' ) Prin9 = 1.if ( icd10d1 EQ 'F30.1' ) Prin9 = 1.if ( icd10d1 EQ 'F31.7' ) Prin9 = 1.if ( icd10d1 GE 'F33.1' AND icd10d1 LE 'F33.2' ) Prin9 = 1.if ( icd10d1 GE 'F33.8' AND icd10d1 LE 'F33.9' ) Prin9 = 1.if ( icd10d1 EQ 'F34.9' ) Prin9 = 1.if ( icd10d1 GE 'F44.5' AND icd10d1 LE 'F52.9' ) Prin9 = 1.if ( icd10d1 GE 'F53.8' AND icd10d1 LE 'F59' ) Prin9 = 1.if ( icd10d1 GE 'F70.1' AND icd10d1 LE 'F99' ) Prin9 = 1.EXECUTE.VARIABLE LABELS Prin9 ' remaining principal diagnoses F00-99 exclude diagnosis above'.
Prin10: Overall diagnoses of mental illness	F00-99	Overall diagnoses of mental illness	COMPUTE Prin10 = 0.if ( icd10d1 GE 'F00' AND icd10d1 LE 'F99' ) Prin10 = 1EXECUTE.VARIABLE LABELS Prin10 ' principal diagnoses of mental illness F00-99.’.
	Prin1-10	Value labels	VALUE LABELS Prin1 Prin2 Prin3 Prin4 Prin5 Prin6 Prin7 Prin8 Prin9 Prin10 0'no' 1'yes'.

The principal diagnosis in Table2 refers to the diagnosis which is chiefly responsible for the hospital admission [11]. ICD-10-AM refers to International Statistical Classification of Diseases and Related Health Problems, Tenth Revision, Australian Modification [20]. In NSW APDC data, the variable ‘icd10d1’ refers to principal diagnosis; ‘icd10d2’ refers to stay diagnosis and ‘icd10d3’ and following refer to other diagnoses. If stay and other diagnoses are recoded into one diagnosis, the command of ‘DO REPEAT’ and ‘END REPEAT’ should be added to the syntax. For example, the variable which includes one-stay diagnosis and 50 other diagnoses for schizophrenia and schizophrenia-like disorder (Other1) is recoded by the syntax: COMPUTE Other1 = 0. DO REPEAT icd10d = icd10d2 TO icd10d52. If (icd10d GE ‘F20’ AND icd10d LE ‘F22.9’) Other1 = 1. If (icd10d GE ‘F24’ AND icd10d LE ‘F29’) Other1 = 1. END REPEAT. EXECUTE. VARIABLE LABELS Other1' stay and other diagnoses of schizophrenia and schizophrenia-like disorder F20-22, F24-29'.

### Aggregate hospital admissions

Hospital admissions need to be summarised into different perinatal periods for some analyses. The following questions are frequently asked: how many hospital admissions in pregnancy or in the first year after birth? how many first hospital admissions during the study period? and how many days of hospital stay in total between pregnancy and the first year postpartum? To answer these questions, some aggregated SVs need to be created. Table3 show the steps and SPSS syntax for summarizing and sequentially ordering the hospital admissions for principal diagnoses of mental illness during pregnancy.

**Table 3:** The steps and SPSS syntax to aggregate hospital admissions for mental illness during pregnancy

Step	**Variable and explanation**	SPSS Syntax
Summarize hospital admissions of all principal diagnoses of mental illness during pregnancy	Select the period of pregnancy and sort record according to hospital admission date.PPN_mum: mother’s Project Person Number AGEAdmMum: mother’s age in days at hospital admission	USE ALL.COMPUTE filter_$ = ( Admmonth > = 13 Admmonth < = 23).VARIABLE LABELS filter_$ ' Admmonth >= 13 Admmonth < = 23 (FILTER)'.VALUE LABELS filter_$ 0 'Not Selected' 1 'Selected'.FORMATS filter_$ (f1.0).FILTER BY filter_$.EXECUTE.SORT CASES BY PPN_mum (A) AGEAdmMum(A).
	Prin10_sum: the total hospital admissions for principal diagnosis of mental illness during pregnancy	AGGREGATE/OUTFILE = * MODE = ADDVARIABLES/BREAK = PPN_mum /Prin10_sum = SUM(Prin10).
Order the principal diagnoses of mental illness during pregnancy in sequence	Select and sort the records during pregnancy	FILTER OFF.USE ALL.SELECT IF (Admmonth > = 13 & Admmonth < = 23).EXECUTE.SORT CASES BY PPN_mum(A) AGEAdmMum(A).
	Morder: The sequent order of mother’s hospital admission	compute Morder = 1.if ( PPN_mum = lag(PPN_mum )) Morder = lag(Morder ) + 1.format Morder (F2).VARIABLE LABELS Morder 'Mother’s hospital admission order (during pregnancy)'.EXECUTE.
	Order the principal diagnosis of mental illness. Prin10order: the order of mother’s hospital admissions for principal diagnosis of mental illness (F00-99) during pregnancy	IF ( Morder EQ 1) #counter = 0.IF (Prin10 EQ 1 AND #counter GE 1) #counter = #counter + 1.IF (Prin10 EQ 1 AND #counter GE 1) Prin10order = #counter.IF (Prin10 EQ 1 AND #counter EQ 0) Prin10order = 1.IF (Prin10 EQ 1 AND #counter EQ 0) #counter = 1.FORMATS Prin10order (F2).VARIABLE LABELS Prin10order 'The order of mother’s hospital admissions for principal diagnosis of mental illness (F00-99) during pregnancy'.EXECUTE.

For the analysis of total length of hospital stay during pregnancy, the duration of each hospital stay during the period is calculated firstly by using maternal age in days at discharge minus maternal age in days at admission. Then the durations of hospital stay during pregnancy are added together by using the function of aggregate data in SPSS (see Table3).

## Discussion

Register-based and routinely collected data are important sources of disease surveillance and epidemiological studies [21,22]. The data sources have been widely utilized in the Nordic countries, Scotland, United Kingdom, United States, Canada and Australia [5,21]. For rare conditions such as birth defects, spinal surgery and arthroplasty, the data provide an effective means for monitoring the rates [23,24]. By compiling more similar data sources from different countries or regions, the study population for the rare diseases increased significantly and the study results became more reliable [24,25]. On the other hand, data linkage provides a way to extend the study field to broader areas and improve the quality of the linked data [2,10,26]. By linking different data sources according to study objectives, study variables can be increased and the completeness of the variable values can be improved significantly [10,26].

A common limitation of registered or routinely collected data is loss of registration or under-reporting. Favourable health was generally more frequent among the registered than the non-registered, and non-registration may lead to bias in analyses of health inequalities [10,27]. In NSW, Aboriginal mothers were less likely to register their births [10]. The magnitude of under-estimation can be estimated by the capture-recapture method [23]. The master dataset provides a platform for creating an SV which can improve the under-estimation [10][25]. The SV is created from linked data and adds value to the data.

Building a master dataset is essential for linked data analysis. The master dataset is useful for data quality improvement. The more data to be linked when building the master dataset, including internal and external datasets, the greater the chance of improving data quality (including consistency and completeness), and the larger the amount of information made available for research. For a mother’s perinatal psychiatric study, the master dataset will be more useful if it includes, in addition to mothers’ and babies’ information, fathers’ data and other data collections such as the national health insurance data (Medicare), RBDM and the hospital- based Emergency Department Data Collection (EDDC). An optimal linked master dataset should cover all life events and health conditions of a study population in the long term.

The techniques for building a master dataset were derived from the current study data which were relatively simple. For very complicated data, such as the Western Australian Data Linkage System (WADLS), which was instigated in 1995 to link up to 40 years of data from over 30 collections for an historical population of 3.7 million, more linking methods were applied [5]. For example, firstly the study records and variables for specific topics were selected from different data sources and then the data were linked [26].

It should be borne in mind when describing rates or risks before birth that gestational age in MDC refers to the time interval from the first day of women’s last menstrual period (LMP) to her baby’s date of birth rather than the interval between conception and date of birth. The conception date is about 14 days after the first day of women’s LMP. Furthermore, gestational age data is provided in completed weeks rather than days. As a result, the shortest time interval before birth should be expressed in weeks.

For more complicated data linkage, building a variable dictionary, including the SV, is very helpful when checking and analysing data.

To provide a comprehensive and representative picture of maternal mental illness before and after birth, some other limitations of linked data should also be considered when interpreting and disseminating the results [28,29]. Patients’ access to health care impacts hospital admission rates. For psychiatric disorders, severity needs to be considered because only mild and severe patients admitted to hospital.

## Conclusion

Linked data preparation including building a master dataset and calculating the SV can improve data quality and enhance data function.

## Abbreviations

APDC, Admitted Patient Data Collection; CHeReL, The Centre for Health Record Linkage; EDDC, Emergency Department Data Collection; ICD, International Classification of Diseases; ICD-10-AM, International Statistical Classification of Diseases and Related Health Problems Tenth Revision Australian Modification; LMP, Last menstrual period; MDC, Midwives Data Collection; NSW, New South Wales; PHDAS, Pharmaceutical Drugs of Addiction System; RCC, Register of Congenital Conditions; SPSS, Statistical Package for the Social Sciences; SV, statistical variable; SVTI, statistical variable of time interval.

## Competing interests

The authors declare that they have no competing interests.

## Authors’ contributions

FX participated in the study design, data analysis and paper writing. LH participated in the study design. MPA participated in diagnoses grouping. EAS participated in the study design and coordinated and supervised the study. All authors read, revised and approved the final version of the manuscript.

## Pre-publication history

The pre-publication history for this paper can be accessed here:

http://www.biomedcentral.com/1471-2288/12/71/prepub
